# Decreased Klotho Expression Causes Accelerated Decline of Male Fecundity through Oxidative Injury in Murine Testis

**DOI:** 10.3390/antiox12091671

**Published:** 2023-08-25

**Authors:** Ya-Yun Wang, Ying-Hung Lin, Vin-Cent Wu, Yu-Hua Lin, Chia-Yen Huang, Wei-Chi Ku, Chiao-Yin Sun

**Affiliations:** 1 Graduate Institute of Biomedical and Pharmaceutical Science, Fu Jen Catholic University, New Taipei City 242, Taiwan; vic0009@gmail.com (Y.-Y.W.); 084952@mail.fju.edu.tw (Y.-H.L.); 2 Taiwan Consortium for Acute Kidney Injury and Renal Diseases (CAKs), Taipei 100, Taiwan; dr.vincentwu@gmail.com; 3 Department of Internal Medicine, National Taiwan University Hospital, Taipei 100, Taiwan; 4 Department of Chemistry, Fu Jen Catholic University, New Taipei City 242, Taiwan; jorgesuperowl@gmail.com; 5 Division of Urology, Department of Surgery, Cardinal Tien Hospital, New Taipei City 231, Taiwan; 6 Gynecologic Cancer Center, Department of Obstetrics and Gynecology, Cathay General Hospital, Taipei 106, Taiwan; bagiao2003@gmail.com; 7 School of Medicine, Fu Jen Catholic University, New Taipei City 242, Taiwan; 089052@mail.fju.edu.tw; 8 Division of Nephrology, Department of Internal Medicine, Keelung Chang Gung Memorial Hospital, Keelung 204, Taiwan; 9 College of Medicine, Chang Gung University, Taoyuan 333, Taiwan

**Keywords:** Klotho, male infertility, antioxidation, lipid peroxidation, glutathione S-transferases

## Abstract

Oxidative stress is the etiology for 30–80% of male patients affected by infertility, which is a major health problem worldwide. Klotho protein is an aging suppressor that functions as a humoral factor modulating various cellular processes including antioxidation and anti-inflammation, and its dysregulation leads to human pathologies. Male mice lacking Klotho are sterile, and decreased Klotho levels in the serum are observed in men suffering from infertility with lower sperm counts. However, the mechanism by which Klotho maintains healthy male fertility remains unclear. Klotho haplodeficiency (*Kl*^+*/*−^) accelerates fertility reduction by impairing sperm quality and spermatogenesis in *Kl*^+/−^ mice. Testicular proteomic analysis revealed that loss of Klotho predominantly disturbed oxidation and the glutathione-related pathway. We further focused on the glutathione-S-transferase (GST) family which counteracts oxidative stress in most cell types and closely relates with fertility. Several GST proteins, including GSTP1, GSTO2, and GSTK1, were significantly downregulated, which subsequently resulted in increased levels of the lipid peroxidation product 4-hydroxynonenal and apoptosis in murine testis with low or no expression of Klotho. Taken together, the loss of one *Kl* allele accelerates male fecundity loss because diminished antioxidant capability induces oxidative injury in mice. This is the first study that highlights a connection between Klotho and GST proteins.

## 1. Introduction

Infertility is a major health issue worldwide; approximately 7% of men suffer from infertility, affecting 10–15% of couples [1]. Moreover, the mean age of paternity has gradually increased over the past four decades. Advanced paternal age leads to decreased traditional semen quality, sperm DNA integrity, and pregnancy rates, further increasing the chances of birth defects and offspring diseases [2]. Oxidative stress (OS), a key factor in the etiology of aging as well as disease, accounts for 30–80% of male infertility cases [3]. OS impairs sperm quality and function and occurs when reactive oxygen species (ROS) production exceeds the scavenging capability [3,4,5]. ROS are critical for cellular survival and function, but excessive ROS levels lead to oxidative modification of essential macromolecules, including lipids, proteins, and DNA, subsequently damaging cells. Antioxidant mechanisms maintain redox homeostasis in multiple organ microenvironments and prevent ROS-induced cellular damage, which further impairs tissue and organ functions [6].

The *Klotho* (*Kl*) gene, an aging suppressor, encodes the α-Klotho protein (subsequently denoted as Klotho), which is mainly expressed in the kidney but is also found in the brain, pancreatic cells, bone, and gonads [7,8,9]. Klotho has multiple functions, such as mineral and vitamin D metabolism, antioxidation, anti-inflammation, and protective effects against cardiovascular and neurodegenerative diseases [10]. Decreased levels of Klotho have been reported in aged human and mouse populations, and Klotho plays a role in the pathogenesis of many diseases [11,12]. In humans, serum Klotho concentration is inversely related to age [13,14]. Patients with chronic renal failure have substantially reduced Klotho [15]. Men with infertility and lower sperm counts have lower serum Klotho levels than those in men with higher sperm numbers [16]. Klotho-deficient mice carrying either hypomorphic or knockout (KO) alleles display a syndrome that manifests several phenotypic features of aging, including early lethality, hyperphosphatemia, renal disease, multisystem defects, and male infertility [7,11]. Moreover, decreased Klotho expression accelerates aging-related disease development in mice. Gao et al. [17] found that the loss of one *Kl* allele deteriorated kidney function in 18-month-old *Kl-*haplodeficient (*Kl^+/−^*) mice because the downregulated expression of glutathione reductase triggered oxidative damage in the renal tubules, which could be mitigated by the in vivo expression of glutathione reductase [17].

Previous studies have shown that Klotho deficiency causes spermatogenic defects in mice [7,11,16]. Hansen et al. [16] suggested that fibroblast growth factor (FGF23) and Klotho signaling may influence gonadal function, in addition to regulating mineral and vitamin D homeostasis. In humans, Klotho levels positively correlate with sperm quality in men with infertility [16]. However, further studies are necessary to fully understand the effects of Klotho on male infertility. In the present study, we aimed to investigate how decreased Klotho expression accelerates the decline in male fertility and the underlying mechanism of spermatogenesis. Klotho-haplodeficient elderly male mice display reduced fecundity compared with that of age-matched controls at 12 months of age. We sought candidates and pathways most affected by the lack of Klotho through an in-depth analysis of the testicular proteome and identified several significantly downregulated members of the glutathione-S-transferase (GST) superfamily, which can protect cells against OS-induced lipid peroxidation [18]. The decreased antioxidation capacity, which consequently resulted in aberrant accumulated lipid peroxidation and germ cell apoptosis, possibly connected the loss of Klotho with the observed infertile phenotypes in both *Kl* KO and elderly *Kl^+/−^* mice. We conclude that Klotho plays a critical role in preventing harmful oxidative effects on spermatogenesis.

## 2. Materials and Methods

### 2.1. Ethics Statement

All animal experiments were performed in accordance with international guidelines and approved by the Institutional Animal Care and Use Committee (IACUC) of Fu Jen Catholic University (Number: A10747). In this study, *Kl^+/−^* (B6;129S5-*Kl^tm1Lex^*/Mmucd, Stock# 011732) (Gene ID: 16591) mice were obtained from the Mutant Mouse Resource & Research Centers (MMRRC) and the coding exons 2 and 3 of *Kl* gene were deleted according to the website information. The genotyping PCR program and primers were performed according to the standard instructions of the MMRRC.

### 2.2. Fertility Assessment

We examined the effect of the loss of one *Kl* allele on male fertility. Two genotype groups, *Kl*^+/−^ and age-matched controls aged 2 or 12 months, were tested. Each group consisted of three male mice. Each male was bred with two 10-week-old WT females, and the endpoint of the mating test was set to the point at which we had gained five litters for each group. We checked the litters daily and counted the pups on the day of birth.

### 2.3. Animal and Histological Analysis

All animal experiments, including mouse genomic DNA extraction, genotyping, and sperm and histological analyses, were performed according to our previous studies [19]. In this study, we analyzed the mice at three ages containing (i) 35-day-old WT and *Kl* KO mice; (ii) 2-month-old WT and *Kl* KO mice; (iii) 12-month-old WT and *Kl^+/−^* mice. At the appropriate time, mice were weighed and anesthetized with an inhaled mixture of isoflurane 2% and oxygen 1 L/min. Blood was collected using the cardiac puncture method. Testosterone (KA0309; Abnova, Taipei, Taiwan), FSH (CEA830Mu; Cloud-Clone Corp, TX, USA) and LH (CEA830Mu; Cloud-Clone Corp) levels were measured using a commercially available ELISA kit. All procedures were performed according to the instructions of the manufacturer and all standards and samples were analyzed in duplicate.

One of the fresh testes was immediately fixed in Bouin’s solution (Sigma-Aldrich, Merck, Darmstadt, Germany) for up to 24 h, and the other was frozen in liquid nitrogen. The Cauda epididymidis was removed and sliced into small pieces in human tubal fluid medium to release sperm and assess the sperm concentration. Sperm was flushed out from the vas deferens to analyze motility and further fixed and smeared on a slide to evaluate morphology. Histological staining (PAS and Hematoxylin, PAS-H) was used to stain paraffin-embedded testicular sections. The stage of seminiferous epithelium cycles was characterized by the acrosome maturation, morphology, and position of differentiating germ cells and was marked in the lumen. The cell composition and epithelium structure of seminiferous tubules were also evaluated.

### 2.4. Electron Microscopy

Ultrastructural examination of the sperm has been previously described [19]. Briefly, sperms were obtained from the epididymides and vas deferens of WT (n = 3) and *Kl^−/−^* mice (n = 3) and fixed with 4% paraformaldehyde and 0.1% glutaraldehyde at 4 °C for 16 h. On the next day, sperm samples were rinsed and exposed to 1% osmium tetroxide following dehydration with graded concentrations of ethanol. Then, sperm samples were embedded in Spurr’s resin kit (cat-14300; EMS, PA, USA) and ultrathin sections (75 nm) were mounted on copper grids. All samples were observed using a transmission electron microscope (JEM-1400; JEOL, Tokyo, Japan).

### 2.5. Nano Scale Liquid Chromatography Coupled to Tandem Mass Spectrometry (Nano LC-MS/MS) and Western Blotting

Total testicular protein was extracted using a lysis buffer (20 mM Tris/HCl (pH = 8), 150 mM NaCl, 5 mM MgCl2, 0.5% Triton-X 100, 10% glycerol, and a protease inhibitor cocktail), and the testicular proteome was analyzed using nano LC-MS/MS on a Dionex Ultimate 3000 RSLC nano system (Thermo Fisher Scientific, Waltham, MA, USA) coupled to an LTQ Orbitrap XL mass spectrometer (Thermo Fisher Scientific) as previously described [20,21]. The protein samples were processed as follows: reduction with dithiothreitol, S-alkylation with iodoacetamide, and digestion with Lys-C and trypsin. After digestion, the peptides were desalted using the SDB-XC StageTip (3M Company, St. Paul, MN, USA) and SCX StageTip (3M Company). In this study, proteomic characterization was performed using three independent batches with duplicate nano LC-MS/MS analyses. Protein identification and label-free quantification were performed using MaxQuant software (version 1.5.3.8) [22] against the SWISS-PROT sequence database (version 2016_05, with 20,207 human canonical protein sequences). A fixed false discovery rate of 1% was adjusted for peptide and protein levels. The results of mass spectrometry proteomics in this study were uploaded to the ProteomeXchange [23] Consortium through the PRIDE partner repository with the dataset identifier PXD039149.

For Western blotting, the experiments were designed as follows: (i) 2-month-old WT versus *Kl* KO group (n = 3 in each group) and (ii) age-matched WT (n = 3) versus 12-month-old *Kl*^+/−^ group (n = 6). Testis lysates were separated by SDS-PAGE and transferred to PVDF membranes, followed by blocking and incubation with primary antibodies at 4 °C overnight. After washing and incubation with HRP-conjugated secondary antibodies, peroxidase activity was detected by chemiluminescence, and band density was quantified using ImageJ 1.54 software (National Institutes of Health, Bethesda, MD, USA). The primary antibodies included those against GSTP1 (15902-1-AP; Proteintech; 1:3000), GSTO2 (14562-1-AP; Proteintech; 1:3000), GSTK1 (A5226; ABclonal; 1:3000), and GAPDH (G8795; Sigma-Aldrich, Merck; 1:10,000).

### 2.6. Bioinformatic Analysis of the Testicular Proteome

After analysis of three independent biological batches that contained three 2-month-old WT and *Kl* KO mice, we classified proteins with an expression cut-off of ±1.5-fold change (*p* < 0.05, Student’s *t*-test) as DEPs. Gene Ontology (GO) and gene functional classification of significantly upregulated or downregulated DEPs were performed using the Database for Annotation, Visualization, and Integrated Discovery (DAVID) functional annotation clustering tool with default settings (https://david.ncifcrf.gov/, accessed on 15 June 2022) [24,25] to characterize the biological processes, molecular functions, and reactomes affected by *Kl* KO. We also used Metascape, a gene annotation and analysis resource, and integrated numerous databases (e.g., GO Biological Processes, Reactome Gene Sets, STRING) (https://metascape.org/, accessed on 15 June 2022) [26] to depict a network model that emphasizes enhanced processes and PPI data. Metascape also provides information on densely connected network components in the PPI network using the MCODE algorithm. Based on the *p*-value, we selected the best-scoring term for further analysis. The genes we selected for further investigation included *Gstp1* (Gene ID: 14870), *Gsto2* (Gene ID: 68214), and *Gstk1* (Gene ID: 76263). Human testis microarray data, including thirteen normal fertile controls, and eight patients with teratozoospermia, were extracted from the published dataset GSE6872 [27] and normalized to average gene expression. 

### 2.7. Immunofluorescence and Immunocytochemistry Analysis

The procedures were previously described [19]. Briefly, paraffin-embedded testis sections (3 μm) were deparaffinized and rehydrated, and antigens were retrieved by boiling in citrate buffer (pH = 6.0) for 10 min. After blocking with 5% normal goat serum, sections were incubated with diluted primary antibody including at 4 °C overnight. For cellular localization of the three chosen candidates, we used a fluorescent Alexa Fluor 488-conjugated secondary antibody to detect and counterstain the sections with 4′,6-diamidino-2-phenylindole (DAPI, Sigma-Aldrich, Merck) and visualized the acrosome with lectin peanut agglutinin (PNA, L-32458; Invitrogen, Thermo Fisher, Waltham, MA, USA). For the expression of 4-HNE (bs-6313R; Bioss Antibodies, Woburn, MA, USA), diaminobenzidine (brown color) was used to detect peroxidase activity according to the manufacturer’s protocol (K5007, Dako Agilent, Carpinteria, CA, USA). The slides were counterstained with hematoxylin, dehydrated, and mounted. Image analysis was performed using FIJI (ImageJ) 2.13.0 software [28]. The experiments were designed as follows: (i) WT (n = 3) versus *Kl* KO (n = 9) at the age of 2 months and (ii) age-matched WT (n = 3) versus 12-month-old *Kl*^+/−^ mice (n = 9). Thirty seminiferous tubules per animal were randomly selected from two sections. The 4-HNE intensity (brown color) and the area of the seminiferous epithelium in each selected tubule were quantified using FIJI. The results are displayed as the brown color intensity per area, and the expression fold-change of 4-HNE in *Kl* KO or *Kl^+/−^* mice was calculated using the mean of WT mice as a reference.

### 2.8. TUNEL Assay

Apoptotic cells in the testes were examined using the DeadEnd Fluorometric TUNEL System (G3250; Promega, Madison, WI, USA). The sections were deparaffinized, rehydrated, and fixed with 4% formaldehyde according to the manufacturer’s instructions. After rinsing in phosphate-buffered saline (PBS), the sections were incubated with 10 μg/mL proteinase K for 10 min. TdT enzyme was then applied and incubated in a dark humidified atmosphere at 37 °C for 1 h. Finally, the slides were immersed in stop solution and counterstained with DAPI and PNA. Full washes in PBS were performed between each step. Quantification of the apoptotic level in the testis was performed. At least 180 round seminiferous tubules in at least three sections from each mouse were counted, and three WT, nine *Kl* KO and nine *Kl^+/−^* mice were examined. In this study, tubules containing at least two or more TUNEL-positive cells were considered positive. We calculated the percentage of apoptotic cells in the positive tubules relative to the total number of seminiferous tubules.

### 2.9. Statistical Analysis

Results are expressed as the mean ± SD and analyzed using Student’s *t*-test. Data were defined as statistically significant when the *p*-value was less than 0.05. An electronic laboratory notebook was not used.

## 3. Results

### 3.1. Decreased Klotho Expression Causes Accelerated Decline in Fecundity

**Figure 1:**
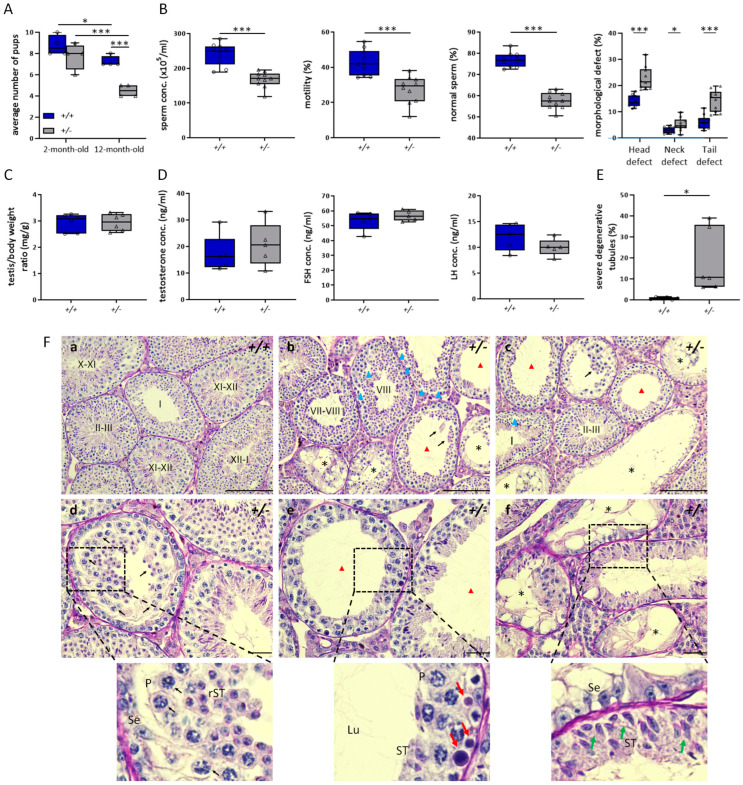
*Klotho* haplodeficiency accelerates loss of male fecundity in 12-month-old mice. (**A**) Comparison of fertile capability between WT (*+/+*) and *Kl^+/−^* (*+/*−) mice at the age of 2 or 12 months. (**B**) Sperm analysis of WT and *Kl^+/−^* mice at the age of 12 months. Concentrations of sperm, percentage of progressively motile sperm, and sperm with morphological defects were analyzed. N = 10 for each genotype. (**C**) Testis (mg)/body weight (g) normalized ratio of WT and *Kl^+/−^* mice. N = 6 per genotype. (**D**) Hormone levels, including testosterone, FSH, and LH of WT and *Kl^+/−^* mice. At least 5 mice per genotype were evaluated. (**E**) Statistical results for testicular histological analysis of WT and *Kl^+/−^* mice. Data exhibit the percentages of severe degenerative tubules. At least 120 tubules were counted at each mouse (N = 6 per genotype). All above data statistics were shown using box and whisker plots and based on * *p* < 0.05 and *** *p* < 0.001 calculated by Student’s *t*-test. Significant differences for the indicated comparison are shown. Circle and triangle represent each individual in the group of WT and *Kl^+/−^* mice, respectively. (**F**) Testicular sections from WT (**a**) and *Kl^+/−^* mice (**b**–**f**) were stained with PAS-H. The stage of spermatogenesis was labeled in the lumen. In (**d**–**f**), the magnified region circled by black dashed box in each image is placed on the bottom. The scale bars represent 200 μm in (**a**–**c**) and 50 μm in (**d**–**f**). Red triangles show thinner seminiferous epithelium, and blue triangles indicate abnormal space or vacuoles. Asterisks mark severe degenerative tubules. Black arrows indicate sloughing germ cells. Red arrows indicate apoptotic-like cells. Green arrows indicate aberrant distribution of germ cells. P, pachytene spermatocyte; rST, round spermatid; ST, elongating spermatid; Se, Sertoli cell.

An association between lower Klotho levels and poor sperm quality in patients with infertility has been reported, although the etiology remains unclear [16]. Based on previous findings, we aimed to clarify whether lower Klotho levels in mice impair male reproductive capacity through an animal model. Hence, we first examined the effect of losing one *Kl* allele on male fertility in mice. We performed fertility assessment and found that the average number of pups produced by 12-month-old *Kl*^+/−^ mice was significantly lower than that of wild-type (WT) mice (4.6 ± 0.55 (*Kl*^+/−^) vs. 7.4 ± 0.55 (WT)), whereas the average litter size did not differ between the 2-month-old *Kl*^+/−^ and WT groups (7.8 ± 1.1 (*Kl*^+/−^) vs. 8.8 ± 0.84 (WT)) (Figure 1A). We ascertained that elderly mice with the loss of one *Kl* allele were fertile but displayed reduced fecundity. 

Next, we further examined the sperm quality and testicular histology of 12-month-old *Kl*^+/−^ mice. The gross appearance and weight of the testes from 12-month-old *Kl*^+/−^ mice were similar to those of age-matched WT mice; however, the sperm quality in *Kl*^+/−^ mice was worse than that of WT mice (Figure 1B,C). We identified obvious reductions in sperm concentration, motility, and normal sperm morphology in *Kl*^+/−^ mice compared with those in WT mice. The percentage of all defects in the head, neck, or tail was much higher in mice with the loss of one allele of the *Kl* gene (Figure 1B). To rule out the potential effects of hormonal alterations that are critical for the maintenance of spermatogenesis, we confirmed no obvious changes in the levels of testosterone, follicle-stimulating hormone (FSH), or luteinizing hormone (LH) between the control and *Kl*^+/−^ mice (Figure 1D). 

Histological examination of the testes showed heterogeneous spermatogenic defects in 12-month-old *Kl*^+/−^ mice. The tubules of the WT testes were filled with fully progressing spermatogenesis with a normal, tight cell distribution (Figure 1(Fa)). In contrast, a number of seminiferous tubules displayed moderate-to-severe hypospermatogenesis and degenerative changes in 12-month-old *Kl*^+/−^ mice (Figure 1(Fb,Fc)). There was abnormal vacuolization in the epithelium (Figure 1(Fb)). Moreover, massive immature germ cells including spermatocytes, round and elongated spermatids were sloughed off from seminiferous tubules (Figure 1(Fd)). Some tubules were thinner due to fewer layers of germ cells and contained apoptotic-like cells (Figure 1(Fe)). In addition, disarranged elongating spermatids irregularly positioned in the basal compartment and loss of spermatocytes were also found in some seminiferous tubules (Figure 1(Ff); lower area). Furthermore, severely degenerative tubules contained only Sertoli cells (Figure 1(Ff); upper area). We also observed that the severity of the spermatogenic impairment was variable among the different *Kl*^+/−^ individuals. A total of 6–39% of severely degenerated seminiferous tubules were found in different 12-month-old *Kl*^+/−^ mice, whereas tubules with atrophic epithelium were barely found in WT mice (Figure 1E).

Consistent with chaotic spermatogenesis, older *Kl*^+/−^ male mice generated abnormal spermatozoa with lower counts, decreased motility, and deformed morphology. We speculated that reduced Klotho expression accelerates the decline in male fertility in *Kl*^+/−^ mice. 

### 3.2. Characteristics of Impaired Spermatogenesis in Kl KO Mice

Next, we investigated the mechanism underlying the accelerated fertility reduction and chaotic spermatogenesis in mice with decreased *Klotho* expression. Spermatogenic defects and disturbed molecular-scale events in mice with a complete lack of Klotho are conceivably more pronounced than those in *Kl* haplodeficient mice. Hence, we analyzed the testicular proteome of Klotho-deficient mice to elucidate the potential mechanisms underlying the role of Klotho in spermatogenic defects.

**Figure 2:**
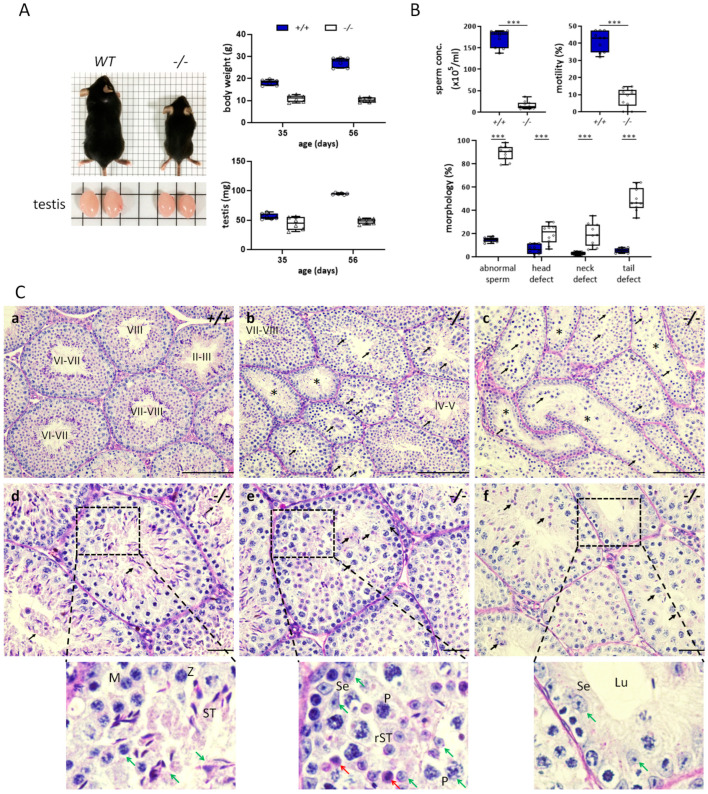
Chaotic and impaired spermatogenesis with high heterogeneities in *Kl* KO mice. (**A**) Gross appearance and testis morphology of WT and *Kl* KO (−*/*−) mice at the age of 2 months. Body and testis weight at postnatal 35 and 60 days. N = 6 for each genotype. (**B**) Sperm analysis of age-matched WT and 2-month-old *Kl* KO mice. Data represent concentrations of sperm, percentage of progressively motile sperm, and each type of sperm morphological defects. All above data were presented using box and whisker plots. N = 10 for each genotype. Significant differences for the indicated comparison are shown (*** *p <* 0.001, analyzed using Student’s *t*-test). Circle and square indicate each individual in the group of WT and *Kl* KO mice, respectively. (**C**) Testicular sections from 2-month-old WT (**a**) and *Kl* KO mice (**b**–**f**) were stained with PAS-H. The stage of spermatogenesis was labeled in the lumen. Magnified views of seminiferous epithelium are the enlarged fields surrounded by the black dashed box in (**d**–**f**). The scale bars represent 200 μm in (**a**–**c**) and 50 μm in (**d**–**f**). Black arrows indicate sloughing germ cells. Asterisks mark atrophy or collapsed tubules with few spermatocytes and Sertoli cells. Red arrows indicate apoptotic-like cells. Green arrows indicate aberrant distribution of Sertoli cells or germ cells. P, pachytene spermatocyte; Z, zygotene spermatocyte; M, meiotic division; rST, round spermatid; ST, elongating spermatid; Se, Sertoli cell; Lu, lumen.

Although previous studies have demonstrated that the loss of Klotho in mice causes infertility, its pathogenesis has not been fully explored [7,16]. Therefore, we first characterized the features of the sperm and testes of *Kl* KO mice in detail. Similar to previous studies [7,16], *Kl* KO mice we used also had a short lifespan of approximately 8–10 weeks and suffered from ill health and growth retardation. Compared to those of age-matched WT littermates, the body and testis weights of *Kl* KO mice stopped increasing weeks 4–5 after birth (Figure 2A). Nevertheless, we observed certain distinct phenotypes in the testes of 2-month-old *Kl* KO mice we used. The overall sperm parameters in *Kl* KO mice were significantly worse than those in control mice (Figure 2B). Histologically, spermatogenic cells in seminiferous tubules of WT mice underwent the full process of germ cell development (Figure 2(Ca)). However, we also found that spermatogenesis of *Kl* KO mice was prominently impacted (Figure 2(Cb,Cc)). The defects in seminiferous tubules included disorganization and massive sloughing of germ cells. Sertoli cells away from their normal position were found in different tubules (Figure 2(Cd,Ce)). Furthermore, more seminiferous tubules containing only a few spermatocytes and Sertoli cells displayed severe atrophy (Figure 2(Cf)).

**Figure 3:**
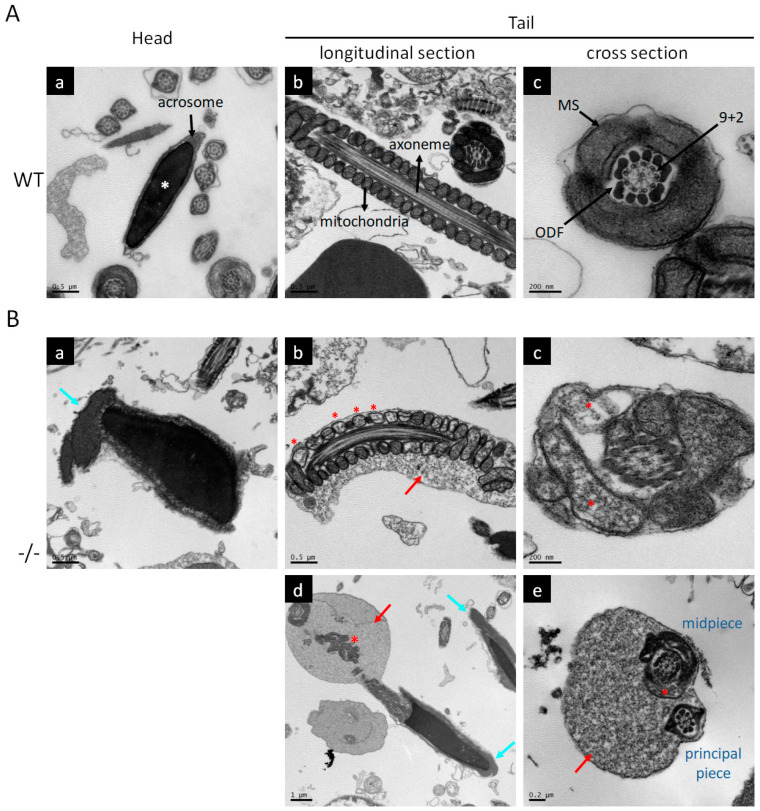
Sperm harbors multiple morphological defects when development under Klotho deficient testicular microenvironment. TEM of WT (**A**) and *Kl* KO (−*/*−, **B**) sperm. Data represent different regions, including the head and longitudinal or cross-sections of the tail. (**A**) Ultrastructure of typical normal sperm. The regular and homogenous head tightly covered by an intact acrosome is marked with a white asterisk (**a**). The flagellum can be divided into four regions: connecting piece, midpiece, principal piece, and end piece. The midpiece comprises a core axoneme structure and outer dense fibers (ODF) surrounded by a mitochondrial sheath (MS) (**b**). Representative “9 + 2” microtubule doublets paired with nine ODF are shown (**c**). (**B**) Most of the sperm from *Kl* KO (−*/*−) mice showed morphological damage in the head and tail. (**a**) Irregular acrosome with membrane deformation (light blue arrows). (**b**,**c**) Numerous abnormal mitochondria in midpiece (red asterisks) and immature sperm with abnormal cytoplasmic droplets (red arrows). (**d**,**e**) Bent-tail structure.

Transmission electron microscopy (TEM) was used to evaluate the sperm ultrastructure in *Kl* KO mice. Sperm from the control mice exhibited regular oval condensed heads and intact tail structures (Figure 3A). In contrast, most sperm from the *Kl* KO group presented multiple abnormalities, including acrosome malformation, residual cytoplasmic droplets, bent tails, and aberrant organization of mitochondria with loss of internal materials (Figure 3B).

Taken together, the homozygosity or heterozygosity of the null allele of *Kl* impairs male fertility in mice. Consistent with the expression of Klotho, damage to spermatogenesis in Klotho-deficient mice was more predominant than in mice with the loss of one *Kl* allele. We reasoned that Klotho levels are critical for maintaining sperm development.

### 3.3. Identification of Factors Related to Klotho Deficiency through Global Proteomic Approach

**Figure 4:**
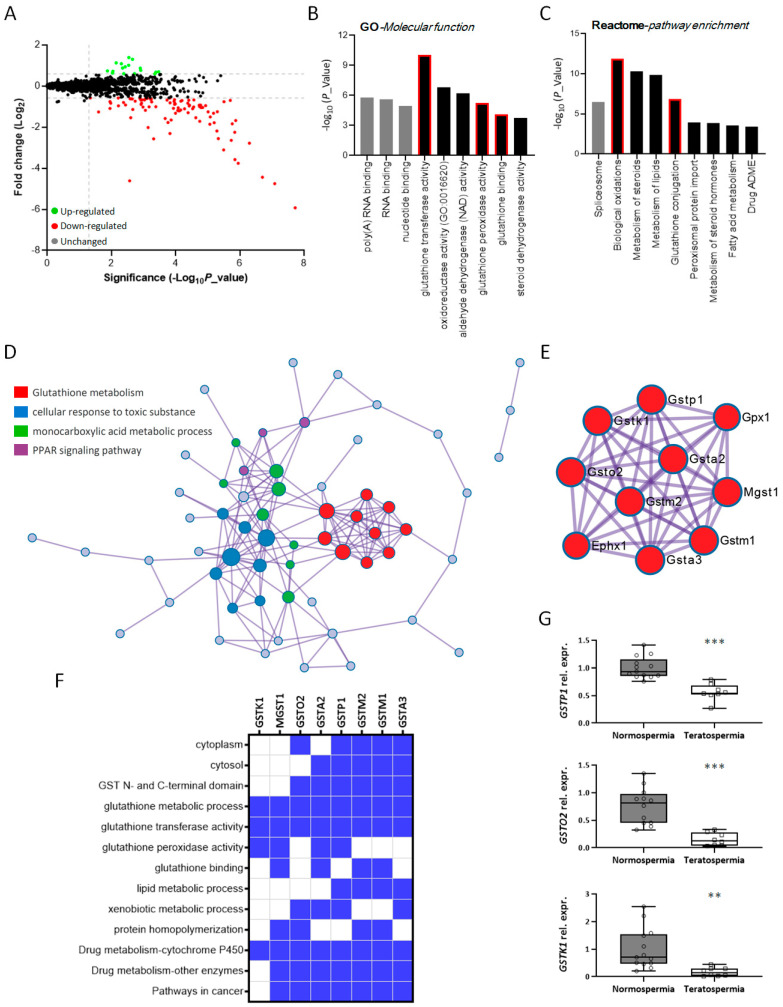
The genes affected by loss of Klotho are mainly involved in antioxidation and associated with human infertility. (**A**) Volcano plots for comparison of all quantified proteins between WT and *Kl* KO mice. Green dots: 17 upregulated genes. Red dots: 91 downregulated genes. Black dots: unchanged genes. N = 3 per genotype. (**B**,**C**) GO analysis of molecular function (**B**) and pathway enrichment (**C**) of a total of 108 differentially expressed proteins in testis. Red boxes mark the function or pathway associated with the antioxidant action of glutathione. (**D**) PPI predicted by Metascape platform which characterizes the most tightly connected network components by the MCODE algorithm. The components of the four significant MCODE modules are highlighted by red, blue, green, or purple nodes. (**E**) The best-scoring GO term by *p*-value is glutathione metabolism (red nodes), and the proteins which construct this sub-network are shown. (**F**) Heat map of the most significant functional related gene cluster analyzed by DAVID. Gene functional classification tool of DAVID summarizes the major biology of the discovered gene group and depicts their annotation. Blue shading indicates that a gene possesses the corresponding property. (**G**) *GSTP1*, *GSTO2*, and *GSTK1* mRNA levels of sperm from fertile individuals (normospermia, N = 13) and infertile men (teratospermia, N = 8). Circle represents each individual data. The available raw data were extracted from dataset GSE6872 and normalized to the average of gene expression. Representative data of each gene display the relative expression of teratospermia to normospermia. ** and *** significant difference at *p* < 0.01 and *p* < 0.001, respectively, analyzed by unpaired *t*-test.

To characterize the functional pathways and molecules that contribute to the potential etiology of male infertility caused by diminished Klotho expression, we compared the testicular proteome between the 2-month-old control and Klotho-deficient groups including three individuals in each group. There were more downregulated genes than upregulated genes in *Kl* KO mice. A total of 108 genes were identified as differentially expressed proteins (DEPs), which consisted of 91 downregulated and 17 upregulated proteins, as depicted in a volcano plot (Figure 4A). To investigate the molecular features of DEPs, gene functional annotations, biological processes, and protein–protein interactions (PPI) were analyzed using different tools to identify enriched terms and pathways. We listed the significant categories according to their *p*-value and noticed the top enriched terms related to glutathione metabolism and antioxidation (Figure 4B,C, highlighted by red boxes). We used the Metascape platform, which applies a Molecular Complex Detection (MCODE) algorithm to analyze and draw densely interconnected regions in the PPI network [26]. The four most significantly enriched MCODE complexes ranked by *p*-value are shown in Figure 4D and Supplementary Table S1, and the components of each MCODE complex-formed subnetwork are shown in Supplementary Figure S1 and Table 1.

Among these four ontologies, glutathione metabolism was the most remarkable term, and eight of the ten proteins involved in this dense subnetwork belonged to the GST superfamily (Figure 4E) and shared common biological characteristics (Figure 4F). Notably, by searching the GEO database, we found that these three genes were strongly correlated with human infertility. After analysis of the published dataset (GSE6872, [27]), the mRNA levels of *GSTP1*, *GSTO2*, and *GSTK1* were remarkably reduced in sperm from infertile men, who had severe sperm morphological defects, compared to sperm from fertile individuals (Figure 4G). To date, only a few functional characterizations of GSTP1, GSTO2, and GSTK1 in male reproduction have been reported. A correlation between *GSTP1* genetic variants and lower sperm quality has been reported [29,30,31]. There is also evidence that the expression of GSTK1 is reduced in low-motility porcine spermatozoa. Moreover, blocking porcine sperm with anti-GSTK1 antibodies led to a poor fertilization rate in an in vitro fertilization assay [32]. GSTO2, which is enriched in the testes and located in the perinuclear theca of murine sperm, participates in nuclear decondensation and is required for complete zygotic development [33]. Because our testicular proteomic data indicated that the loss of Klotho probably compromised the antioxidation process, which may be due to decreased GST protein levels, we further examined the potential effects of the three selected candidates, GSTP1, GSTO2, and GSTK1, on spermatogenesis.

### 3.4. Klotho Depletion Interferes with Oxidative Balance through Attenuating the Protein Levels of GSTs

**Figure 5:**
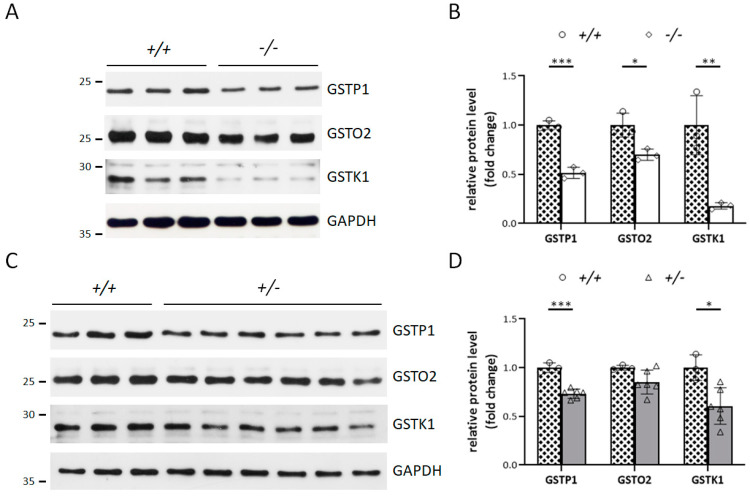
Validation of the expression of the chosen downregulated GST proteins on the testes from Klotho deficient and 12-month-old *Kl^+/−^* mice. Representative Western blotting images and quantitative analysis of GSTP1, GSTO2, GSTK1, and GAPDH in: (**A**,**B**) 2-month-old WT (*+/+*) and *Kl* KO (−*/*−) mice. N = 3 for each genotype; (**C**,**D**) 12-month-old WT (n = 3) and *Kl^+/−^* (*+/*−) mice (n = 6). GADPH protein was used as a loading control. The expression of GSTP1, GSTO2, and GSTK1 was normalized to GAPDH protein, and the expression fold change of the selected three proteins in *Kl* KO or *Kl^+/−^* mice was calculated using the mean of the WT mice as a reference. All data are displayed as the mean ± standard deviation (SD), and any significant difference compared with the WT group is indicated by asterisks. * *p <* 0.05, ** *p <* 0.01, *** *p <* 0.001, analyzed by unpaired *t*-test.

Next, we investigated whether the selected candidates, GSTP1, GSTO2, and GSTK1, played important roles in the observed infertility caused by decreased Klotho expression. First, Western blotting was conducted to verify the effect of the loss of one or both *Kl* alleles on the expression of these GSTs in the testes. In *Kl* KO testes, the levels of GSTP1, GSTO2, and GSTK1 proteins were reduced by approximately 0.48-, 0.3-, and 0.82-fold, respectively (Figure 5A,B). Similar diminishing trends in these three GSTs were also observed in elderly Klotho-haplodeficient mice. In the 12-month-old *Kl*^+/−^ group, the levels of GSTP1, GSTO2, and GSTK1 were also decreased by approximately 0.27-, 0.15-, and 0.4-fold, respectively, compared with those in age-matched controls (Figure 5C,D). Consistent with the Klotho levels, the reduced levels of GSTP1, GSTO2, and GSTK1 expression in the *Kl* KO group were more pronounced than those in the 12-month-old *Kl*^+/−^ group. 

**Figure 6:**
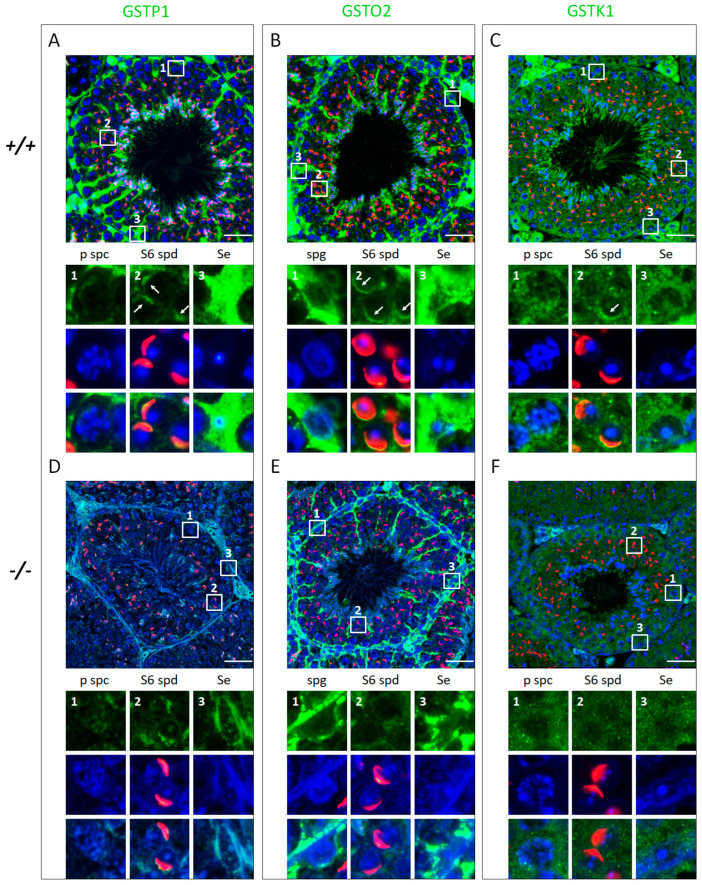
The wide expression and potential multiple functions of GSTP1, GSTO2, and GSTK1 during spermatogenesis. Immunofluorescence staining for GSTP1 (**A**,**D**), GSTO2 (**B**,**E**), and GSTK1 (**C**,**F**) counterstained with DAPI (blue) and PNA (acrosome marker, red) in the testicular sections of 2-month-old WT (*+/+*) and *Kl* KO (−*/*−) mice. White boxes with numbers highlight the expression of these three proteins in different cell types; magnified images are shown in the lower panels. Images of the WT and *Kl* KO testicular sections stained with the same antibody were acquired with the same exposure time. Scale bars represent 50 μm. White arrows indicate colocalization with the acrosome. p spc, pachytene spermatocytes; S6 spd, round spermatids at step 6; spg, spermatogonia; Se, Sertoli cells.

Since the expression of these three GSTs was affected by Klotho levels in the testes, we performed immunofluorescence staining of testicular sections to visualize the cell types expressing GSTP1, GSTO2, and GSTK1. Immunostaining of 2-month-old WT testicular sections revealed that both GSTP1 and GSTO2 were strongly detected in Sertoli cells. In premeiotic germ cells, higher GSTO2 intensities were detected in spermatogonia, and lower GSTP1 or GSTK1 signals were observed in pachytene spermatocytes. Furthermore, all proteins colocalized with the acrosome in round spermatids during spermiogenesis (Figure 6A–C). To compare the changes of the three GST candidates in the testicular cells between *Kl* KO and WT mice, we preferentially examined the seminiferous tubules with minor defects that remained of different types but had fewer germ or somatic cells to severely degenerated tubules in *Kl* KO testes. In contrast, the fluorescence intensities of these three GSTs were generally diminished in whole testicular sections of *Kl* KO mice, in addition to the GSTO2 signals in Sertoli cells (Figure 6D–F). These results revealed that GSTP1, GSTO2, and GSTK1 may protect against OS in testicular cells, including somatic and germ cells, to maintain redox balance during spermatogenesis.

During spermatogenesis, male germ cells undergo a high rate of cell division and mitochondrial activity, which is linked to the production of numerous ROS, probably resulting in harmful effects on spermatogenesis [34,35]. Germ cells are more susceptible to OS than somatic cells for several reasons. First, they interact closely with ROS-producing phagocytic Sertoli cells [36]. Second, the germ cell plasma membrane is comprised of abundant long-chain polyunsaturated fatty acids (PUFAs), which are important targets of OS-induced lipid peroxidation that eventually generates common metabolites such as 4-hydroxynonenal (4-HNE) [37,38,39]. As the most toxic lipid aldehyde, 4-HNE can form adducts with proteins and frequently alters protein function, DNA damage, and apoptosis [40,41,42]. Various enzymatic pathways participate in 4-HNE clearance to protect cells. The major metabolic pathway of 4-HNE is conjugation with glutathione mediated by GSTs, which contain glutathione peroxidase and transferase activity, hence protecting against membrane lipid peroxidation under the OS cascade [18,42,43,44,45].

Based on the aforementioned knowledge, we suggest that lower levels of GST proteins, such as GSTP1, GSTO2, and GSTK1, lead to the inappropriate accumulation of 4-HNE and subsequent apoptosis in the testis with decreased Klotho. Hence, we examined the levels of 4-HNE and apoptosis in the testes of *Kl* KO and elderly *Kl^+/−^* mice using immunohistochemical staining and a terminal deoxynucleotidyl transferase-mediated dUTP nick end labeling (TUNEL) assay, respectively.

**Figure 7:**
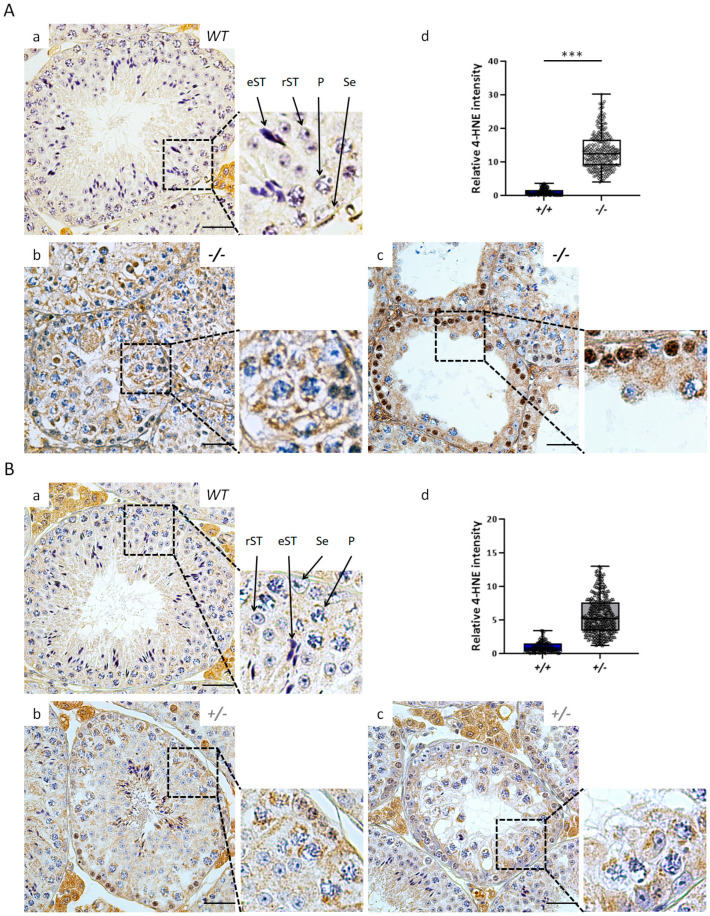
The increased 4-HNE level in testes with lower or no expression of Klotho. The presence of 4-HNE was confirmed by immunohistochemistry of testicular sections counterstained with hematoxylin (blue) and further quantified. (**A**) 2-month-old WT (*+/+*, **a**) versus *Kl* KO (−*/*−, **b**,**c**); (**B**) 12-month-old WT (*+/+*, **a**) and *Kl^+/−^* (*+/*−, **b**,**c**) mice. The insets show enlarged boxed areas. Scale bars represent 50 μm. Whisker-box blots (**d**) depict the quantification of 4-HNE intensity within the seminiferous tubules (n = 3 for each WT group and n = 9 for *Kl* KO and *Kl^+/−^* groups. Thirty tubules per mouse were examined). The total 4-HNE intensity (brown color) within each tubule was analyzed using FIJI (ImageJ) 2.13.0 software and normalized to the area of the seminiferous epithelium. The relative intensity of 4-HNE in *Kl* KO or *Kl^+/−^* mice was calculated using the mean of WT mice as a reference. Any significant difference compared to the WT group is indicated by asterisks. *** *p <* 0.001, analyzed by unpaired *t*-test. P, pachytene spermatocytes; rST, round spermatids; eST, elongated spermatids; Se, Sertoli cells.

**Figure 8:**
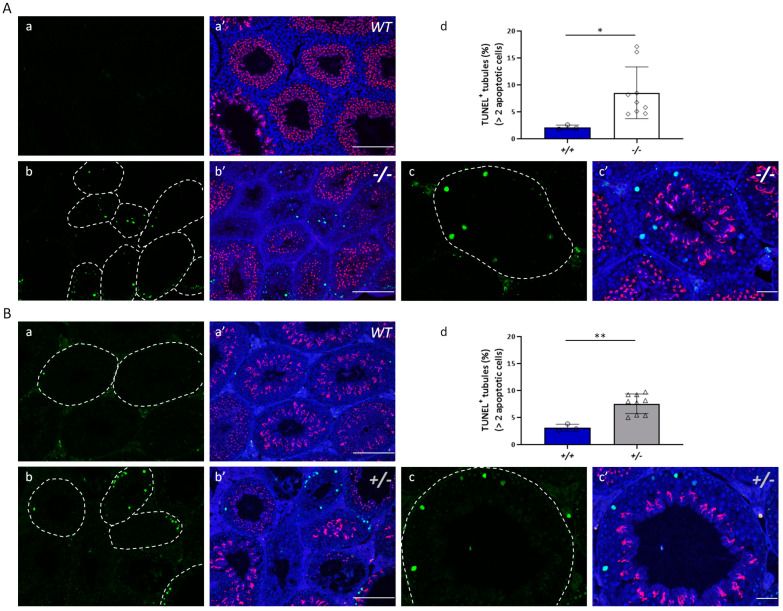
Oxidative injury caused by accumulated 4-HNE leads to apoptosis of germ cells in mice with the decreased level of Klotho. TUNEL assay was used to evaluate the level of apoptosis in the testes. (**A**) 2-month-old WT (*+/+*, **a**) vs. *Kl* KO (−*/*−, **b**,**c**); (**B**) 12-month-old WT (*+/+*, **a**) and *Kl^+/−^* (*+/*−, **b**,**c**) mice. The white dotted line indicates seminiferous tubules with TUNEL signals (green). Nuclei and acrosomes were stained with DAPI (blue) and PNA (red), respectively, and displayed in merge images (**a’**, **b’**, and **c’**). The scale bars represent 200 μm in (**a’**,**b’**) and 50 μm in (**c’**). Percentage of the apoptotic level (**d**) which is represented as the percentage of positive tubules relative to the total number of tubules. Tubules containing two or more apoptotic cells were defined as positive tubules, and at least 180 seminiferous tubules were examined in each mouse. Data are presented as the mean ± SD. (n = 3 for each WT group and n = 9 for *Kl* KO and *Kl^+/−^* groups). * *p <* 0.05, ** *p <* 0.01 compared to the WT group.

After quantification of the 4-HNE staining intensity, an abundance of 4-HNE was found in the seminiferous tubules of both *Kl* KO and 12-month-old *Kl*^+/−^ mice (Figure 7). Compared with those of 2-month-old WT mice (Figure 7(Aa)), more intense 4-HNE signals were observed in the spermatogonia, pachytene spermatocytes, and Sertoli cells of *Kl* KO mice (Figure 7(Ab,Ac)), especially in severely degenerated tubules (Figure 7(Ac)). We also uncovered a similar trend toward increased germ and Sertoli cell staining in the seminiferous tubules of *Kl*^+/−^ testes compared with those in age-matched WT testes at 12 months of age (Figure 7B). These data were consistent with an increased apoptotic population in testes with lower or deficient Klotho expression (Figure 8). TUNEL staining indicated significantly increased levels of cell apoptosis not only in *Kl* KO (8.53 ± 4.79% (*Kl* KO) vs. 2.11 ± 0.43% (WT), Figure 8A) but also in 12-month-old *Kl*^+/−^ mice (7.17 ± 2.08% (*Kl*^+/−^) vs. 3.09 ± 0.65% (WT), Figure 8B), compared with those in age-matched WT mice, respectively.

Taken together, these results confirm our hypothesis that impaired antioxidant capacity due to downregulated expression of GSTs results in the aberrant accumulation of 4-HNE, which consequently induces apoptosis in murine germ cells with diminished Klotho expression.

## 4. Discussion

**Figure 9:**
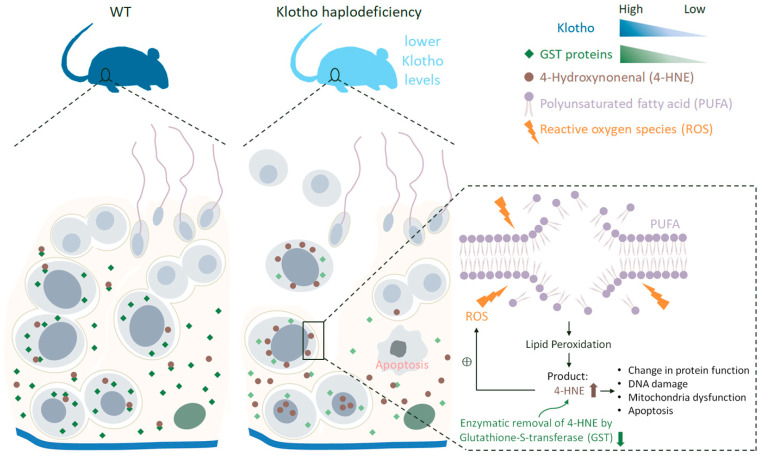
Summary graphic model of the role of Klotho in the pathogenesis of the male infertility.

In this study, we revealed the antioxidation of Klotho in male reproduction. Elderly mice lacking one *Kl* allele exhibit subfertility due to aberrant spermatogenesis and lower sperm quality. To characterize the events underlying perturbed spermatogenesis in testes with decreased Klotho expression, we analyzed the testicular proteome and found that Klotho participates in maintaining a balanced oxidation status by regulating GST protein levels in the testicular microenvironment. Decreased Klotho levels accelerate the decline in male fecundity because the abnormal accumulation of 4-HNE produced by OS-induced lipid peroxidation in germ and somatic cells impairs spermatogenesis (Figure 9). 

Although mice with Klotho deficiency are infertile, and an association between serum Klotho levels and human infertility has been reported [16], the etiology remains unclear. Previous studies have indicated that *Kl* KO mice exhibit male infertility due to complete spermatogenic arrest at the spermatocyte stage [7,11]. However, the testicular histology of global *Kl* KO mice generated by Hansen et al. [16] showed that germ cell development in some seminiferous tubules was arrested at the spermatocyte stage but complete spermatogenesis was observed in more tubules (histological images not shown in the study) [16]. Compared with the results reported by Hansen et al. [16] we identified similar but certain different spermatogenic defects in our *Kl* KO mice. Hansen et al. [16] also revealed that the sperm count in 2-month-old fertile *Kl*^+/−^ mice was lower than that in WT mice; however, we found that the fecundity of *Kl*^+/−^ mice decreased at 12 months of age. We speculate that the phenotypic diversity in *Kl* KO testes observed in these studies may be caused by different knockout strategies. Klotho-deficient mice used by Tsujikawa et al. and Hansen et al. [16] carried different knockout alleles with deletions in exons 1 and 2, respectively [11,16,46]. Exons 2 and 3 were the target-deleted regions of *Kl* KO mice in our study. As mentioned above, the effects of deleting different coding regions of *Kl* on the function, structure, and stability of the Klotho protein probably led to the phenotypic heterogeneity in the testes observed in different strains of *Kl* KO mice.

*Kl*, an anti-aging gene, has been well studied for its multiple functions and exerts pathogenic roles in many diseases, such as osteoporosis, chronic kidney disease (CKD), cancer, and infertility. The Klotho protein mainly consists of a transmembrane segment and two extracellular domains and exists in membrane-bound and soluble forms. The membrane-bound form is an obligate coreceptor of FGF23 that cooperatively regulates phosphate and vitamin D metabolism. The soluble Klotho form is produced by the proteolytic cleavage of the membrane-bound form (shedding). In addition, it is the major form discovered in the circulation and acts as an endocrine hormone [7,11,47]. Most aging-like organ dysfunctions can be rescued by restoring mineral homeostasis and vitamin D toxicity in *Kl* KO mice. The infertile phenotypes found in the gonads of mice lacking Klotho may be secondary effects of hyperphosphatemia and hypervitaminosis D on the disruption of normal endocrine secretion from the hypothalamus, pituitary gland, or adrenal gland. [48,49,50]. Nevertheless, Hansen et al. [16] suggested that Klotho may play distinct roles in spermatogenesis because *Kl*-haplodeficient mice have decreased sperm counts but normal serum mineral levels [16]. Consistent with these insights, we found that 12-month-old *Kl*^+/−^ mice with comparable serum testosterone concentrations had significantly lower sperm quality and chaotic spermatogenesis. Furthermore, *Kl*^+/−^ elderly mice exhibited diminished antioxidant capacity owing to lower expression of GST proteins. We presume that Klotho exerts antioxidative functions in the testis. To our knowledge, this is the first study to elucidate the relationship between Klotho and GST.

Antioxidation is a critical function of Klotho and is mediated through the nuclear factor erythroid 2-related factor 2 (Nrf2) and FoxO forkhead transcription factor antioxidant pathways [51,52]. Klotho protects the renal, cardiovascular, and neuronal systems against harmful effects of OS through the activation of Nrf2, a transcription factor that regulates the responses to OS and toxins [53,54,55]. Overexpression of Klotho through adenoviral delivery in diabetic mice upregulated Nrf2 activation and ameliorated oxidative stress and podocyte apoptosis, which were accompanied by mitigated renal damage [55]. The fertility of *Nrf2* KO male mice progressively deteriorated with increasing age because of the accumulation of seminiferous tubule defects until the age of 6 months. Some of the degenerative alterations found in the tubules of *Nrf2* KO mice were similar to the testicular phenotypes observed in our mice, which lacked either one or both Kl alleles. Moreover, Nakamura et al. [48] demonstrated the relationship between Nfr2 and GSTs. The mRNA levels of *Gsta3* and *Gstm1* were substantially lower in 2-month-old *Nrf2 KO* testes than those in WT testes, although *Gstp* mRNA levels were not affected [56]. Bartolini et al. showed that Nrf2 upregulation increased the expression of GSTP (also known as GSTP1) [57]. Through in-depth analysis of the testicular proteome, we found that the decreased Klotho could affect the oxidation status as the levels of GSTP1, GSTO2, and GSTK1 were significantly decreased in somatic and germ cells. Hence, we suggest that Klotho may affect GST protein expression by regulating Nrf2 activity in the testes. However, this hypothesis needs to be further verified. A limitation of the present study is that we did not ascertain the mechanism by which Klotho regulates GST protein expression in the testes.

At least 40% of male patients with infertility are diagnosed with idiopathic disease, and OS is one of the strongly implicated etiologies [1,4,5]. The myriad PUFAs in the plasma membrane of germ cells are vulnerable targets for ROS damage, which results in lipid peroxidation chain reactions and consequently generates byproduct 4-HNE [36,39]. 4-HNE serves as a signaling molecule but becomes toxic at higher levels because of its ability to initiate the OS cascade. Another reason is that the formation of 4-HNE-adducts on proteins and DNA adversely affects protein function, causes DNA damage, and induces apoptosis [42]. Furthermore, 4-HNE is harmful to sperm function, causing impairments in motility, morphology, acrosome reaction, and gamete interactions [41,58,59]. Conjugation with glutathione, mediated by GSTs, is the main detoxification mechanism of 4-HNE in most tissues.

GST proteins have multiple functions, including defense mechanisms against OS, cellular detoxification of xenobiotics and toxins, and regulation of male fertility [18]. To the best of our knowledge, most studies have focused on the antioxidant protective effects of GSTs on spermatozoa during their transition through the male reproductive tract and seminal plasma. Llavanera et al. [18] summarized the secretion of GST proteins in the male reproductive tract and the expression of GSTs in rodent testes [18]. However, whether GSTs modulate redox balance during spermatogenesis remains unclear. *Gstm2* RNA is strongly detected in type A and B spermatogonia and dramatically decreases during spermatogenesis [60]. Our data revealed that GST proteins, including GSTP1, GSTO2, and GSTK1, were widely expressed in Sertoli and germ cells. We also found excessive accumulation of 4-HNE and a higher percentage of apoptosis in the germ cells of both *Kl* KO and 12-month-old *Kl*^+/−^ testes. Taken together, we suggest that GSTP1, GSTO2, and GSTK1 maintain a balance in the oxidation status of the mouse testicular microenvironment.

The potential role of GSTs in fertilization has been explored in several studies. Petit et al. found that GSTM3 is localized to the acrosomal region of human sperm and interacts with human zona pellucida (ZP) glycoproteins such as ZP3 and ZP4 [61]. Hamilton et al. [25] found that GSTO2 facilitates sperm nuclear decondensation during fertilization in mice. Inhibition of GSTO2 causes abnormal embryonic development [33]. In the present study, GSTP1, GSTO2, and GSTK1 were found to be localized in the acrosome of round spermatids during spermiogenesis. In addition to GSTO2, GSTP1 and GSTK1 are also likely to play different roles in acrosome biogenesis and fertilization.

Klotho performs numerous functions in biological processes and pathologies, and its levels diminish with age in many diseases. Accordingly, it has great potential for application in the diagnosis and management of diseases and in Klotho-based therapy. Klotho expression is nearly completely suppressed in many cancer types and is positively correlated with a better prognosis [10]. The serum concentration of Klotho in patients begins to decrease at an early stage in CKD; hence, Klotho may be a predictor of renal function [62]. Furthermore, both male and female patients with CKD exhibit remarkable fertility defects due to multiple pathogenic factors, including uremic toxins, alterations in reproductive hormone profiles, sexual dysfunction, chronic inflammation, and OS [63]. Men with advanced CKD have impaired sperm quality, including lower sperm concentration, motility, and normal morphology [63,64]. Our results demonstrate that Klotho is involved in the maintenance of oxidative balance through the regulation of GST protein levels in the testes. This may contribute to impaired spermatogenesis and poor sperm quality in patients with CKD. For therapeutic effects, Klotho supplementation to restore its levels in patients with kidney diseases may improve not only renal function but also fertility. Further studies investigating the underlying mechanisms by which Klotho functions in spermatogenesis are required to provide further evidence for preclinical studies.

## 5. Conclusions

In this study, we identified several GST proteins, possibly linking the lack or lower of Klotho expression with the observed spermatogenic defects, and revealed the potential antioxidant role of Klotho in male fecundity. Understanding the effects of Klotho on male reproduction is beneficial for the clinical management and treatment of male infertility.

## Figures and Tables

**Table 1:** The downregulated proteins involved in the top four significant MCODE modules.

Gene Symbol	Protein Name	Log_2_ FC	Majority Protein IDs
**MCODE_1**	glutathione metabolism		
Ephx1	Epoxide hydrolase 1	−2.783	E9PWK1; Q9D379; Q6PEV0; Q8K2W5
Gsta3	Glutathione S-transferase A3	−2.644	P30115; K9JA27; Q9DCU1; A0A087WQI6
Gstm1	Glutathione S-transferase Mu 1	−1.788	P10649; A2AE89; F6WHQ7
Mgst1	Microsomal glutathione S-transferase 1	−1.544	D3YU60; Q53ZD4; E9QJW0; Q91VS7; A0A0N4SV17; D3YVR3
Gstk1	Glutathione S-transferase kappa 1	−1.508	Q9DCM2
Gstm2	Glutathione S-transferase Mu 2	−1.104	P15626; D3YX76; Q7M0F4
Gsta2	Glutathione S-transferase A2	−0.840	P10648; D3Z6A6; D3YZV3
Gsto2	Glutathione S-transferase omega-2	−0.738	Q9D2J1; A0A494BAY2; Q9D2S1; Q8BW12; Q8K2Q2; A0A494BB82
Gstp1	Glutathione S-transferase P 1	−0.707	A0A494B908; A0A494BAW2; P19157
GPX1	Glutathione peroxidase 1	−0.681	A0A0A6YY34; A0A0A6YVV2; P11352
**MCODE_2**	monocarboxylic acid metabolic process		
Acox3	Peroxisomal acyl-coenzyme A oxidase 3	−3.109	Q3TAW3; E9Q296; Q8C178; Q9EPL9
Akr1cl	Aldo-keto reductase family 1, member C-like isoform 1	−1.861	Q9D5U2; G3XA14; Q80W25; Q3UXL1
Scp2	Non-specific lipid-transfer protein	−1.218	P32020-2; P32020
Aldoc	Fructose-bisphosphate aldolase C	−1.185	P05063
Acox1	Peroxisomal acyl-coenzyme A oxidase 1	−1.008	Q9R0H0-2; Q9R0H0; A2A848; Q8C168; Q8BW35
Akr1d1	3-oxo-5-beta-steroid 4-dehydrogenase	−0.969	Q8VCX1
Acaa1a	3-ketoacyl-CoA thiolase A, peroxisomal	−0.957	Q921H8; Q3UPU8; H3BJZ9; H3BKL5; Q8BLD7
Akr1c12	Aldo-keto reductase family 1 member C12	−0.933	Q9R0M7; Q9JLI0; Q91X42
**MCODE_3**	cellular detoxification		
Gpt2	Alanine aminotransferase 2	−1.778	Q8BGT5
Cat	Catalase	−1.258	Q3UF58; Q91XI2; Q8C6E3; Q542K4; Q3UZE7; Q3TVZ1; P24270
Aldh1a7	Aldehyde dehydrogenase, cytosolic 1	−1.254	B2RTL5; O35945
Ttr	Transthyretin	−1.140	Q5M9K1; P07309; Q9D6A4
Aldh1a1	Retinal dehydrogenase 1	−1.110	P24549
Aldh1b1	Aldehyde dehydrogenase X, mitochondrial	−0.844	Q9CZS1
Tpi1	Triosephosphate isomerase	−0.679	P17751; P17751-2; H7BXC3
Aldh2	Aldehyde dehydrogenase, mitochondrial	−0.610	Q544B1; Q3UJW1; Q3U9J7; P47738; A0A0G2JEU1; Q3U6I3; Q3TVM2
**MCODE_4**	PPAR signaling pathway		
Fabp3	Fatty acid-binding protein, heart	−2.254	Q5EBJ0; P11404
Me1	Malic enzyme; NADP-dependent malic enzyme	−0.703	Q99LF5; Q921S3; P06801; Q3TQP6
Apoa1	Apolipoprotein A-I	−0.678	Q3V2G1; Q00623; Q58EV2

## Data Availability

All data generated during this study are included in the article.

## Supplementary Materials

The following supporting information can be downloaded at: https://www.mdpi.com/article/10.3390/antiox12091671/s1, Figure S1: The network of the top four enriched MCODE complexes ranked by *p*-value. Table S1: Description of the four significant MCODE modules from the PPI network.
