# Supplementary figures and images for: Decreased Klotho Expression Causes Accelerated Decline of Male Fecundity through Oxidative Injury in Murine Testis
Source: Antioxidants (Basel). 2023 Aug 25;12(9):1671. doi: 10.3390/antiox12091671 (PMC10526093; doi:10.3390/antiox12091671)

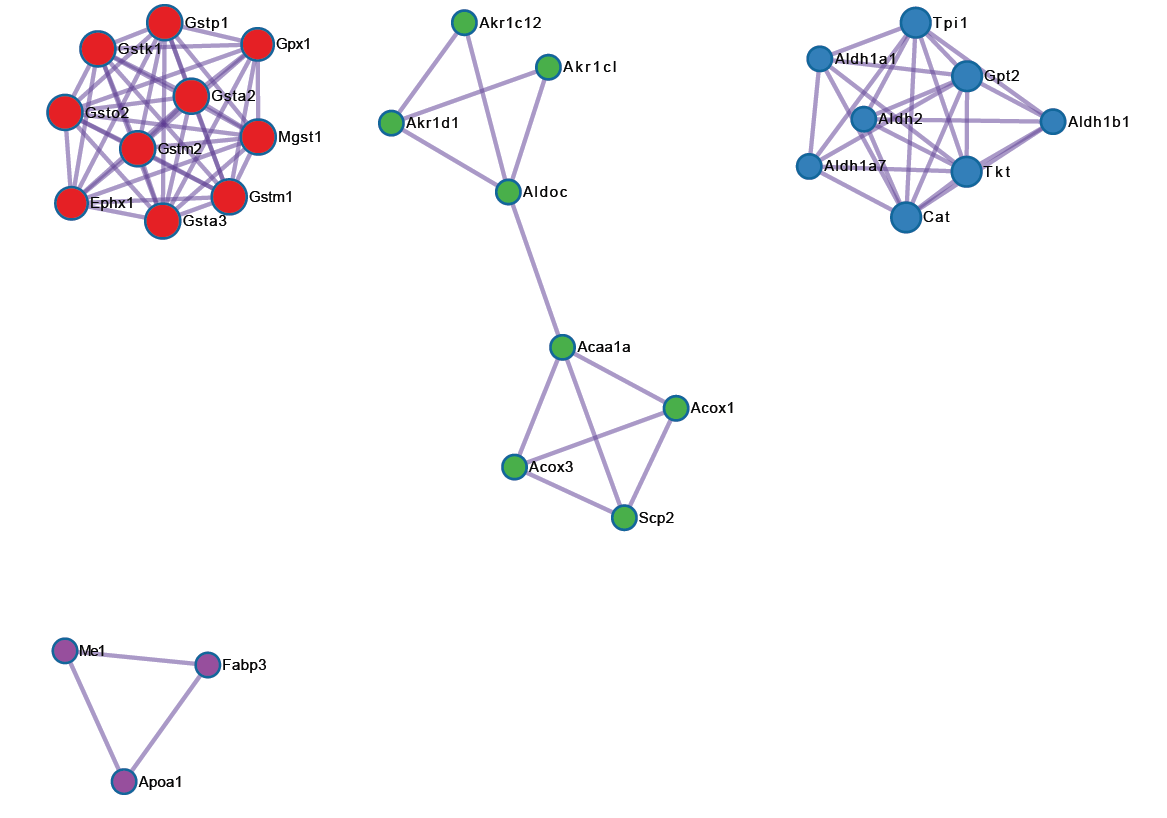


**Figure S1.** The network of the top four enriched MCODE complexes ranked by p-value.

Supplement: Supplementary file 1 [file antioxidants-12-01671-s001.zip › Supplementary Fig S1 - +▌+s.docx]
